# Adherence to Interferon β-1b Treatment in Patients with Multiple Sclerosis in Spain

**DOI:** 10.1371/journal.pone.0035600

**Published:** 2012-05-16

**Authors:** Oscar Fernández, Javier Agüera, Guillermo Izquierdo, Javier Millán-Pascual, Lluis Ramió i Torrentà, Pedro Oliva, Javier Argente, Yasmina Berdei, Jose Maria Soler, Olga Carmona, Jose Maria Errea, Jordi Farrés, on behalf of the Group on Adherence to IFNb-1b in Spain

**Affiliations:** 1 Hospital Universitario Carlos Haya de Málaga, Málaga, Spain; 2 Hospital Universitario Reina Sofía Córdoba, Spain; 3 Hospital Virgen de la Macarena, Sevilla, Spain; 4 Hospital La Mancha Centro, Alcázar de San Juan, Spain; 5 Hospital Josep Trueta, Girona, Spain; 6 HUC de Asturias, Oviedo, Spain; 7 Hospital Puerta del Mar, Càdiz, Spain; 8 Hospital de Salamanca, Salamanca, Spain; 9 Hospital de Sant Joan de Déu de Manresa, Manresa, Spain; 10 Hospital de Figueres, Figueres, Spain; 11 Hospital de Barbastro, Barbastro, Spain; 12 Bayer Hispania, Barcelona, Spain; Institute Biomedical Research August Pi Sunyer (IDIBAPS) - Hospital Clinic of Barcelona, Spain

## Abstract

**Background:**

Adherence to interferon β-1b (INFβ-1b) therapy is essential to maximize the beneficial effects of treatment in multiple sclerosis (MS). For that reason, the main objectives of this study are to assess adherence to INFβ-1b in patients suffering from MS in Spain, and to identify the factors responsible for adherence in routine clinical practice.

**Methodology/Principal Findings:**

This was an observational, retrospective, cross-sectional study including 120 Spanish patients with MS under INFβ-1b treatment. Therapeutic adherence was assessed with Morisky-Green test and with the percentage of doses received. The proportion of adherent patients assessed by Morisky-Green test was 68.3%, being indicative of poor adherence. Nevertheless, the percentage of doses received, which was based on the number of injected medication, was 94.3%. The main reason for missing INFβ-1b injections was forgetting some of the administrations (64%). Therefore, interventions that diminish forgetfulness might have a positive effect in the proportion of adherent patients and in the percentage of doses received. In addition, age and comorbidities had a significant effect in the number of doses injected per month, and should be considered in the management of adherence in MS patients.

**Conclusion/Significance:**

Among all the available methods for assessing adherence, the overall consumption of the intended dose has to be considered when addressing adherence.

## Introduction

Multiple Sclerosis (MS) is a chronic progressive disease of the central nervous system with a very heterogeneous clinical course [1]. Clinically, MS usually initiates with an acute inflammatory demyelinating episode (clinically isolated syndrome – CIS), suffering later on new clinical episodes turning to relapsing-remitting MS (RRMS). Many of these patients develop into a secondary-progressive MS (SPMS) in approximately 10 years time [2], [3]. Alternatively, 10% of patients may suffer from a primary progressive form of the disease (PPMS) since onset.

Although MS is still an incurable disease, several randomized clinical trials have demonstrated the benefits of disease modifying drugs (DMD) in reducing the relapse rate, the rate of disability progression, and the magnetic resonance imaging (MRI) outcomes [4]–[12]. The Multiple Sclerosis Therapy Consensus Group (MSTCG) [13] recommends early initiation of immunotherapy with any of these DMDs after the first episode suggestive of MS, because of its efficacy in preventing and delaying relapses [6].

Immunomodulatory treatments are long-term therapies that might be difficult to sustain by MS patients over a long period of time. Then, poor adherence is a critical issue that compromises the effectiveness of DMD therapies. According to the World Health Organization (WHO), adherence is the extent to which a person's behavior corresponds with agreed recommendations from a health care provider [14]. Overall, adherence to long-term therapies for chronic illnesses in developed countries averages 50% [14], and it accounts for 60% to 90% of patients with MS [15]–[22]. Most studies conducted to assess adherence in MS patients have been focused on discontinuation rates [15]–[19], [21], [22], being side effects, perception of lack of efficacy, missed disease improvement, or disease worsening the main reasons to stop treatment [23]. In these cases, discontinuation is most likely to occur within the first six months of treatment [24]. Besides discontinuation, adherence might be focused on prescribed treatment regimen. To date, the available information to this definition of adherence is limited to the studies of Tremlett et al. [20] and Devonshire et al. [25] in which adherence was defined as not missing a single DMD injection.

As the identification of the factors that affect adherence is the first step in improving therapeutic compliance, and therefore, in obtaining the maximum benefits of long-term treatment [25], this study achieved to assess the adherence to prescribed INFβ-1b treatment in patients with MS in Spain.

## Methods

### Objectives

This study aimed to assess adherence to prescribed INFβ-1b treatment in patients with MS in Spain, to identify the main reasons for non-adherence, and to identify factors that may be responsible for poor adherence in routine clinical practice.

### Participants

Eligible patients were those with diagnosis of CIS, RRMS or SPMS, and those on INFβ-1b treatment at recommended doses for at least six months before the study enrollment. In addition, eligible patients must have retrieved medication from the hospital pharmacy, and attend to routine follow-up visits. This study included patients who consecutively attended to 13 Spanish centers between October 2008 and February 2009 and met all the inclusion criteria. The number of patients who were enrolled in the study was the planned per-protocol. In addition, patients were recruited by order of attendance to their physician's to avoid selection bias

### Study design

This is a multicentre, observational, retrospective, cross-sectional study with a single visit to assess the adherence of MS patients to INFβ-1b treatment in Spain.

### Treatment

Interferon β-1b (250 µg/ml) was injected subcutaneously (SC) every other day (EOD) during at least six months before to the study enrollment.

### Variables and measurements

The main variable of the study was self-reported adherence to INFβ-1b in the previous four weeks assessed by Morisky-Green (MG) test [26]. This test was selected because of its reliability (61%), and because it was validated to the Spanish language [27]. In addition, MG test assess attitudes towards treatment. According to MG test, patients were fully adherent when correctly answered the four-item questionnaire: Had you ever forgotten to inject your medication? (NO); are you rigorous in regard to your injection hours? (YES); do you skip your injection hours when you are feeling well? (NO); and when you feel worse due to the medicine, do you skip your injection? (NO).

Adherence was also evaluated by means of the percentage of doses received that was calculated by dividing the number of self-administered injections by the number of injections that should have been administered in the previous four weeks.

In addition, the number of injections dispensed by the hospital pharmacy during the previous six months, and the number of forgotten injections, as well as reasons for forgetting them during the previous month were assessed.

Other variables recorded were demographic data (age, sex, marital status, education level, employment status), MS clinical data (MS diagnosis, age at first episode, age at diagnosis, time since last relapse), clinical setting characteristics (existence of MS unit), MS treatment (time on INFβ-1b therapy), general health status (existence of anxiety/depression (yes/no), regular fatigue (yes/no), comorbidities, and concomitant treatments), and adverse events. The Expanded Disability Status Scale (EDSS) [28] were used to assess the degree of disability caused by MS.

### Statistical analysis

Descriptive statistical analyses were performed by means of the software SAS® 9.1.3. Continuous variables were described by means of their average and standard deviation (SD), while categorical variables were described by counts (N) and frequencies. The relationship between baseline variables (demographic, clinical, and clinical setting characteristics) and adherence was analyzed. For that reason, patients were divided into two categories: adherent and non-adherent. The differences between study groups were done by chi-square test for categorical variables, and t-Student test or analyses of variance (ANOVA) for continuous variables at a significant level p<0.05.

Additionally, the effect of baseline variables (predictor variables) on the number of injections administered during the previous month was studied by means of a univariate regression model. Afterwards, the significant predictor variables were used in a final multivariate regression model.

### Ethics

The local ethical review board of Hospital Universitario Carlos Haya de Málaga approved the protocol of the study according to the principles of the Declaration of Helsinki. All patients had received detailed information on the study and had provided their written informed consent prior to their inclusion.

## Results

### Baseline demographic and clinical data

Baseline demographic and clinical data are summarized in Table 1. A total of 120 patients (64.2% females) were enrolled at 13 Spanish centers. Overall, patients had been suffering from MS for an average time of 11.7±8.9 years, and most of them were suffering from RRMS.

**Table 1:** Demographic, clinical and clinical setting characteristics at baseline.

Demographics	N = 120
Age, years (mean ± SD)	41.2±11.2
Marital Status [N (%)]	
Single	37 (30.8)
Married	75 (62.5)
Divorced	6 (5.0)
Widow/er	2 (1.7)
Education level [N (%)]	
Primary	44 (36.7)
Secondary	45 (37.5)
University	31 (25.8)
Employment status [N (%)]	
Active worker	40 (33.3)
Disabled	25 (20.8)
Housewife	23 (19.2)
Pensioner	14 (11.7)
Student	9 (7.5)
Unemployed	9 (7.5)

MS clinical factors	N = 120
MS diagnosis [N (%)]	
RRMS	94 (78.3)
SPMS	22 (18.3)
CIS	4 (3.3)
Age at 1^st^ episode (mean ± SD)	29.5±9.1
Age at diagnosis (mean ± SD)	32.2±9.8
Time since last episode (mean ± SD)	3.1±3.5
EDSS score (mean ± SD)	2.7±2.1
Anxiety/depression present [N (%)]	26 (21.7)
Regular fatigue present [N (%)]	56 (47.1)
Comorbidity present [N (%)]*	38 (31.7)
Hypertension [N (%)]	3 (8.1)

Clinical setting characteristics	N = 13
Centers with MS unit [N (%)]	6 (46.2)
Neurologists exclusively dedicated to MS [N (%)]	2 (15.4)
Centers with nurses exclusively dedicated to MS [N (%)]	10 (76.9)
Number of check-up during the first year of treatment (mean ± SD)	3.1±1.4
Number of check-up during the second year of treatment (mean ± SD)	2.6±0.8

RRMS: recurrent-remittent MS; SPMS: secondary-progressive MS; CIS: clinically isolated syndrome.

* Comorbidities are listed in Table 6.

### Treatment

Patients were treated for an average of 5.3±4.5 years, and titration was used in 75% of them. INFβ-1b was used at the standard dose of 250 µg/ml EOD, 85.8% of the patients self-injected INF-1β medication, usually using an auto-injector (91.7%), all of them had received previous training by a nurse, and 52.9% kept regularly a record of the date of injections.

### Adherence to INFβ-1b

According to MG test, the proportion of patients adherent to treatment was of 68.3% (82/120 patients). Among these patients, 79.2% (95/120) of them had never forgotten to take any injection, and among the remaining adherent patients (20.8% [25/120]), 56.5% had forgotten only one injection). Among the 31.7% (38/120) of non-adherent patients,13.3% (16/120) did not administer their injections at the indicated hours, 2.5% (3/120) decided not to inject because of feeling better, and 5.8% (7/120) decided not to inject because of feeling worse.

The percentage of doses received in the last month was 94.3%, the average number of administered injections in that period was 14.2±2.1 (being 15 the expected doses), and 12.5% of the patients administered less than 13 injections. The percentage of doses received was higher among adherent patients according to MG (96.2% vs. 90.2%, p = 0.0089), and only 28.0% of adherent patients according to MG administered less than 15 injections.

Besides the MG test and the percentage of doses received, the number of injections dispensed by the hospital pharmacy during the six months period before the assessment was used as an adherence measurement. The mean number of injections dispensed by the hospital pharmacy was similar for all the six months, being 89.7±52.6 the total number of injections dispensed at the end of the follow-up period. Nevertheless, 64.5% (60/93) of patients had kept medication at home, being non-adherent according to MG test criteria, the patients who tend to store more injections (9.5±9.9 injections vs 5.7±6.1 injections).

### Reasons for missing INFβ-1b injections

The main reasons for missing INFβ-1b injections are showed in Table 2. Our results showed that the primary reason for missing doses were forgetting injections (64.0%), followed by patient's decision (24.0%) and side effects (20.0%). None of the patients missed injections due to perception of lack of efficacy.

**Table 2:** Reasons for missing INFβ-1b injections (N = 25).

	N (%)
Forgetting injection	16 (64)
Own will decision	6 (24)
Adverse events	
Fatigue	2 (8)
Flu symptoms	1 (4)
Headache	1 (4)
Infections	1 (4)
Avoid disturbances in social commitments	1 (4)

### Factors affecting adherence to INFβ-1b

Our results demonstrated that none of the demographic and clinical variables at baseline had a significant relationship with the adherence to therapy, neither MS phenotype, nor time since first and last episodes, or EDSS scores. Regarding clinical setting characteristics, the proportion of adherent patients as assessed by MG test and the percentage of doses received did not differ between patients who were followed in centers with MS unit and those who did not (Table 3).

**Table 3:** Adherence to INFβ-1b treatment according to the existence of an MS unit.

Existence of MS unit			
	Yes (N = 67)	No (N = 53)	p-value
Morisky-Green Test [N (%)]			0.2714*
Adherent	43 (64.2)	39 (73.6)	
Non-adherent	24 (35.8)	14 (26.4)	
Adherence rate (mean ± SD)	93.33±11.72	95.47±11.90	0.3262†

* Chi-square test.

† ANOVA.

General health status variables, INFβ-1b treatment characteristics, concomitant treatments, and adverse events did not show any significant relationship with the proportion of adherent patients (Table 4). Regarding adverse events, patients were more adherent when they rarely or sometimes suffered from pain at the injection site than when they suffered from pain more frequently (p = 0.0486)[Table 4].

**Table 4:** General health status variables and adverse events according to adherence as assessed by MG test.

	Adherent	Non-adherent	p-value*
**General health status**, N	82	38	
Presence of depression/anxiety [N (%)]	14 (17.1)	12 (31.6)	0.0728
Fatigue [N (%)]	36 (44.4)^a^	20 (52.6)	0.4042
**Presence of comorbidities** [N (%)]	23 (28.0)	15 (39.5)	0.2107
**Concomitant treatments** [N (%)]	41 (50.0)	19 (51.4)	0.8914
**INFβ-1b treatment characteristics**			
Time on INFβ-1b treatment, years (mean ± SD)	6.1±5.8	6.0±4.4	0.9273†
**Presence of adverse events** [N (%)]	62 (75.6)	31 (81.6)	0.4664
Pain at the injection site, N	42	21	0.0486
Rarely/sometimes [N (%)]	27 (64.3)	8 (38.1)	
Very often/Always [N (%)]	15 (35.7)	13 (61.9)	
Redness at the injection site, N	42	21	0.3534
Rarely/sometimes [N (%)]	27 (57.4)	11 (45.8)	
Very often/Always [N (%)]	20 (42.6)	13 (54.2)	
Inflammation at the injection site, N	26	19	0.0789
Rarely/sometimes [N (%)]	19 (73.1)	9 (47.4)	
Very often/Always [N (%)]	7 (26.9)	10 (52.6)	
Fever, N	18	10	0.4543
Rarely/sometimes [N (%)]	12 (66.7)	8 (80.0)	
Very often/Always [N (%)]	6 (33.3)	2 (20.0)	
Muscular pain, N	22	13	0.6891
Rarely/sometimes [N (%)]	15 (68.2)	8 (61.5)	
Very often/Always [N (%)]	7 (31.8)	5 (38.5)	
Shivering, N	27	11	0.6106
Rarely/sometimes [N (%)]	20 (74.1)	9 (81.8)	
Very often/Always [N (%)]	7 (25.9)	2 (18.2)	
Headache, N	30	13	0.3131
Rarely/sometimes [N (%)]	21 (70.0)	11 (84.6)	
Very often/Always [N (%)]	9 (30.0)	2 (15.4)	

a N = 81.

* Chi-square test.

† ANOVA.

### Multivariate analysis

The results of the multivariate regression model showed that, among all the baseline variables, only age and the presence of concomitant diseases were significant predictors of the number of monthly injections (Table 5). According to this model, young patients and patients who suffered from comorbidities (see Table 6) were more likely to self-inject more doses, and, therefore to be more adherent.

**Table 5:** Multivariate regression model for the number of injections administered during the previous month.

	N	Estimator	Standard Error	p-value
Age	120	−0.03742	0.01686	0.0283
Concomitant diseases		0.89561	0.40723	0.0298

**Table 6:** Comorbidity profile of the patients suffering from MS (N = 37).

	N (%)
Hyperlipoproteinemia	8 (21.6)
Hypertension	5 (17.5)
Rheumatoid arthritis	1 (2.7)
Diabetes mellitus	4 (10.8)
Migraine	3 (8.1)
Cardiac disorder	3 (8.1)
Trigeminal neuralgia	2 (5.4)
Thalassemia	2 (5.4)
Asthma	2 (5.4)
Atopy	1 (2.7)
Allergic conjunctivitis	1 (2.7)
Vitamin B_12_ deficiency	1 (2.7)
Ménière's disease	1 (2.7)
Epilepsy	1 (2.7)
Fibromialgy	1 (2.7)
Vertebral artrosis	1 (2.7)
Trigeminal neuralgia	1 (2.7)
Hypothyroidism	1 (2.7)
Urinary tract infection	1 (2.7)
Venous insufficiency	1 (2.7)
Mastectomy	1 (2.7)
Spinal disc herniation	1 (2.7)
Osteoarthritis	1 (2.7)
Osteoporosis	1 (2.7)
Sciatica	1 (2.7)
Sleep paralysis	1 (2.7)
Deep venous trombosis	1 (2.7)
Fibromialgy	1 (2.7)
Dysthymia	1 (2.7)
Hypophysiary bening tumor	1 (2.7)

Patients might have more than one comorbidity.

### Safety

Most patients (77.5%) experienced at least one adverse event during the previous month, mainly redness (59.1%) or pain (52.5%) at site of injection. Other adverse events such as inflammation at the injection site, muscular pain, headache, fever, and shivering were less frequent.

## Discussion

The results of this retrospective cross-sectional study showed that 68% of patients adhered to INFβ–1b treatment, both to the dose and time recommended by their physician. Although the minimum desirable levels of adherence in MS are currently unknown, our results were below the 80% and 95% considered as satisfactory in other chronic diseases [29]. Nevertheless, the percentage of doses received yielded better results and reached 94% with 88% of patients injecting almost 87% of the intended dose.

To date, several studies have been conducted on adherence to DMD therapies for MS [15]–[25]. However, most of them have been focused on discontinuation rates rather than on adherence to prescribed doses [15]–[19], [21], [22]. To our knowledge, two studies have assessed adherence to an intended number of doses [20], but only the study of Devonshire et al. [25] evaluated adherence for a month in patients on therapy for at least six months before the study inclusion. Although the results of these authors were similar to ours (70% of patients were adherent to INFβ-1b treatment), their definition of adherence was based on missing a single dose without considering either adherence to timing or the overall consumption of the prescribed doses. In the other study, in which adherence to an intended number of doses was assessed [20], adherence was defined also as missing a single dose but for a longer period of time (six months) and in patients on therapy only for the month before the study inclusion. The results of these authors showed very low adherence rates (only 25% of patients were adherent to INFβ-1b), but their findings were not comparable to ours because patients are more likely to miss doses and withdraw treatment during the first six months of treatment [25]. Nevertheless, these authors found that the overall consumption of the prescribed doses was good (88% of patients injected at least 80% of the intended dose). In fact, our results also showed that, although a proportion of patients were considered as non-adherent, their overall consumption of INFβ-1b was good (above the 80% of the prescribed dose). Interestingly, our results also showed that a proportion of patients considered as adherent by Morisky-Green test injected less than 15 injections per month. This results show up that indirect measures of adherence such as questioning the patient, although being relatively easily to use, can be susceptible to misinterpretation and tended to overestimate the patient's adherence [29].

Besides MG test and the percentage of doses received, we used the number of injections dispensed by the hospital pharmacy to measure adherence. Among indirect methods to assess adherence to medication regimens, rates of refilling prescriptions in a closed pharmacy system are an alternative accurate measure of adherence provided that refills are measured at several points of time [29]–[31]. We found, however, that the monthly total number of injections dispensed by the hospital pharmacy was not such an accurate expected measure to assess therapeutic adherence. In fact, although the amount of injections dispensed by the hospital pharmacy was similar during the six months follow-up period of the study, 65% of patients kept doses at home.

In this study, the primary reason for missing doses was forgetfulness (64%), followed by patient's decision (24%). The recently reported results of Devonshire et al. [25] also found that forgetting to administer injections was the most common reason for missing injections. Some studies [25], [24] suggested that forgetting injections may be related to the complexity of treatment regimens, lower cognitive function or other neuropsychological problems such as depression. In our study, anxiety/depression might have an effect on adherence because the proportion of patients with those conditions was slightly higher among non-adherent patients than among adherent patients. Taking into account that patients with MS are likely to suffer from depressive disorders [32] and that depression negatively affects adherence, some authors have suggested that routinely screening for depression and early treatment are interventions that might enhance adherence to treatment [25], [33], [34]. Besides the screening and treatment of these diseases, other interventions focused on diminishing forgetfulness like regimen simplification or medication reminders might also improve adherence.

We found that, although more than half of our patients suffered from pain or redness at the injection site, these injection-related events did not lead to skipping doses. Nonetheless, some studies have found that injection-related issues such as adverse events at the injection site, injection anxiety, or needle phobia usually led patients to skip injections or to stop treatment [18], [25], [34]. For that reason, interventions focused on making self-injection easier, and the future availability of a non-injected therapy might improve adherence [25], [34].

Patient's subtype of MS influences the likelihood of adherence to therapy in terms of discontinuation [25]. In fact, the study of Río et al. [19] found that the proportion of patients stopping DMD was significantly higher in patients with SPMS than in those with RRMS. Although our study failed to find any relationship between MS subtype and the number of injected medication, it may be due to the small number of patients with SPMS and CIS that were enrolled in the study.

An increased probability of adherence has also been associated to the existence of a MS unit [25]. Moreover, thorough care and service at the beginning of treatment, as well as educational interventions to correct unrealistic improvement expectations, indirectly increased adherence mainly because of the decreasing in the dropout rates [19], [35]. Nevertheless, our findings did not show the expected trend because the proportion of patients who had been treated in centers with MS unit, and who potentially might have received better care and information on adherence and treatment outcomes, had similar adherence outcomes than those treated in centers without MS unit.

Our results demonstrated that none of the demographic and clinical variables at baseline had a significant relationship with the adherence to therapy, neither MS phenotype, nor time since first and last episodes, or EDSS scores. Regarding clinical setting characteristics, the proportion of adherent patients as assessed by MG test and the percentage of doses received did not differ between patients who were followed in centers with MS unit and those who did not Finally, our multivariate analysis showed that only age and comorbidities were significant predictors of the overall number of injections administered in the previous month. Although Reynolds et al. [22] demonstrated that patients below 34 years-old and those with cardiovascular comorbidities were more likely to discontinue DMD therapies, the effect of these variables on the overall number of injected doses or on the likelihood of missing doses have never been found. In fact, only Tremlett et al. [20] and Devonshire et al. [25] assessed predictors of adherence (defined as missing a single dose), and found that the level of education, duration of MS, time since last relapse, clinical setting characteristics, history of missed doses, and alcohol consumption predicted adherence in patients with MS. Our findings, thus, pointed out that besides these variables, age and comorbidities also have to be taken into account in the management of adherence in patients with MS.

In summary, the results of this study highlighted the importance of the methodology used to assess adherence to treatment in the management of patients with MS in clinical practice. In fact, although we found that the proportion of adherent patients was not optimal, the overall consumption of the intended dose was. Therefore, the number of injected doses or, alternatively, the number of missed doses had to be taken into account when predicting the consequences of poor adherence.
