# Correction: Adherence to Interferon β-1b Treatment in Patients with Multiple Sclerosis in Spain

**DOI:** 10.1371/annotation/35c70f05-8483-4310-aa2a-4665f4842129

**Published:** 2012-06-05

**Authors:** Oscar Fernández, Javier Agüera, Guillermo Izquierdo, Javier Millán-Pascual, Lluis Ramió i Torrentà, Pedro Oliva, Javier Argente, Yasmina Berdei, Jose Maria Soler, Olga Carmona, Jose Maria Errea, Jordi Farrés, on behalf of the Group on Adherence to IFNb-1b in Spain

There were errors in the published author names. The correct author by-line is: Oscar Fernández, Eduardo Agüera, Guillermo Izquierdo, Jorge Millán-Pascual, Lluis Ramió i Torrentà, Pedro Oliva, Joaquin Argente, Yasmina Berdei, Jose Maria Soler, Olga Carmona, Jose Maria Errea, Jordi Farrés, on behalf of the Group on Adherence to IFNb-1b in Spain.

The correct citation is: Fernández O, Agüera A, Izquierdo G, Millán-Pascual J, Ramió i Torrentà L, et al. (2012) Adherence to Interferon β-1b Treatment in Patients with Multiple Sclerosis in Spain. PLoS ONE 7(5): e35600. doi:10.1371/journal.pone.0035600 

